# How big is the medical writing industry? Why it matters

**DOI:** 10.1093/heapro/daaf146

**Published:** 2025-09-05

**Authors:** Maud Bernisson, Sergio Sismondo

**Affiliations:** Institute for Science in Society, Radboud University, Postbus 9102, 6500 HC Nijmegen, Netherlands; Laboratoire Interdisciplinaire Sciences Innovations Sociétés (LISIS), CNRS, Université Gustave Eiffel, 5 Boulevard Descartes, 77420 Champs-sur-Marne, France; Department of Philosophy, Queen’s University, Kingston, ON, K7L 3N6, Canada

**Keywords:** medical writing industry, scientific communication, medical education and communication companies, pharmaceutical companies, industry-funded research

## Abstract

Medical writing is a key element in pharmaceutical companies’ efforts to shape the relevant medical science literature. As part of what is called ‘publication planning’, medical writing can influence the knowledge base on which prescribers make decisions, and can build specific claims in targeted sales efforts. Most publication planning is done by hired medical education and communication companies (MECCs), with the rest done by other commercial entities, such as units of pharmaceutical companies or of contract research organizations, that provide essentially the same services as MECCs. Here we provide an estimate of the number of MECCs and comparable entities contributing to the medical science literature in English. To identify these companies, we collected data from Web of Science (858 named firms from 20 498 papers mentioning medical writing assistance), LinkedIn (410 company profiles), and Google and DuckDuckGo (68 company websites). After removing duplicates and false positives, we found 1148 MECCs and other comparable entities providing medical writing services. More than 50% of Web of Science papers that acknowledged medical writing support are sponsored by only ten pharmaceutical companies. Most of the remaining papers in our database are sponsored by other pharmaceutical, device, and biotechnology companies. This study likely undercounts MECCs, because it depends on some level of transparency in publications or other leakage of information. Our combining multiple sources for the data should limit the undercount of MECCs. The study does not identify MECCs that work exclusively in languages other than English.

Contribution to Health Promotion

- The number of MECCs and similar entities offering medical writing services is substantial, and yet this industry is often overlooked—if not unknown—by researchers, health professionals, patients and policymakers.
- The firms we catalogued earn their medical writing revenue by working not for individual authors but for industry, whose overriding interest is profit.
- Results suggest an infrastructure of influence, allowing industry to quietly shape the medical literature, and thus quietly shape the evidence base on which many medical decisions are made.
- Impacts can be particularly harmful when this literature conveys health information that supports the industry’s products, with little consideration to their consequences on public health.

## INTRODUCTION

If you occasionally scan the acknowledgements sections of peer-reviewed papers in medical journals, you may have noticed sentences that read something like:

Medical writing support was provided by [name of a person], Ph.D., of [name of a company], funded by [name of a pharmaceutical company].

Such gestures at transparency are anything but transparent. As we describe below, the involvement of paid medical writers is frequently connected to industry efforts to shape and circulate medical information and opinions. ‘Medical writing support’ is often less innocuous than it sounds.

Here we provide a sense of how much industry-funded medical writing there is. To do so, we provide an estimate of the number of medical education and communication companies (MECCs), and similar entities contributing to the medical science literature in English—though some of these companies also work in other languages. These companies offer medical writing services and other scientific communication services; that is, they develop and deliver tailored content for healthcare professionals and patients. Products may include slide decks and posters for conferences and other events, manuscripts for peer-reviewed publications—on which we focus here—as well as related aspects of the organization of industry influence on medical science and opinion.

Medical writing is a key element of ‘publication planning’ in the pharmaceutical, medical device, and biotechnology industries (Sismondo 2018)—since it is the largest of these three, we will refer only to the ‘pharmaceutical industry’. Publication planning is the development and execution of marketing plans via scientific and educational communication. It includes the description and production of articles and presentations for target journals, conferences, and educational events. It attempts to systematically map out, prepare, and use medical publications to market products, including peer-reviewed journal articles, conference presentations, posters, and more. Publication planning allows client companies to publish medical science that promotes their products or can be used to promote them (Bernisson and Sismondo 2024).

Ideally, for the companies, publication plans would have wide sweeps, encompassing the development of a lexicon and key arguments and claims, the multiplication of results by lumping and splitting data (Melander *et al*. 2003), all incorporated in scientific communications such as publications. Eventually, circulating these outputs also requires the enrolment and rewarding of what are usually referred to as ‘key opinion leaders’ or simply ‘KOLs’. KOLs are physicians and researchers hired to speak or write on behalf of pharmaceutical companies (Scher and Schett 2021); they are assumed, or sometimes simply positioned, to have substantial influence on health professionals, researchers and patients to whom they communicate.

MECCs and medical writers are crucial to the success of publication planning because of their skill in communicating their clients’ preferred messages. Yet to be effective, the extent of pharmaceutical companies’ control over the messages, and thus their hiring of MECCs, cannot be easily visible (e.g. Fugh-Berman 2010; McHenry 2010; Sismondo 2018). The result is often a kind of ‘ghostwriting’, which occurs when pharmaceutical companies hire professional writers to produce or substantially contribute to medical journal articles and other medical content, published under the names of independent researchers. The ghostwriters are either un- or under-acknowledged: We should understand ghostwriting as allowing for a range of transparency and ghostliness (e.g. Gøtzsche *et al*. 2009).

Although industry-funded articles in medical journals are often ‘authored’ by academics, authorship does not necessarily represent significant contributions to the research, design, or writing of the articles (e.g. PLoS Medicine Editors 2009). The authors of ghostwritten journal articles written for a pharmaceutical company typically include the company’s preferred KOLs (e.g. Bernisson and Sismondo 2024). In industry articles, authors typically make only modest contributions, contributions that rarely meet the spirit of authorship requirements (Jureidini *et al*. 2016). These commissioned articles often have somewhat ghostly writers, as well as ghostly researchers, statisticians, and planners (Gøtzsche *et al*. 2007). The ghostwritten literature might be as much as 11% of English biomedical publications (Flanagin *et al*. 1998) or as much as 40% of the core clinical publications on recently approved drugs (Sismondo 2007).

Researchers have found various windows into the activities and practices of ghostwriting. To detail particular instances of ghostwriting, most studies have relied on documents made available through litigation (e.g. Fugh-Berman 2010, McHenry 2010, Amsterdam and McHenry 2012, Jureidini *et al*. 2016, Ross *et al*. 2008, Bernisson and Sismondo; 2024). Other researchers have built on insider knowledge as medical writers or publication planners (Matheson 2008, Logdberg 2011), or on insider knowledge as KOLs who were to be authors on ghostwritten papers (Healy 2004, Fugh-Berman 2005). Some researchers have done more ethnographic research, attending publication planning conferences and seminars, and collecting publications and advertisements aimed at insiders (Sismondo 2018). These studies make no attempt to establish the size or scope of the medical writing industry, which is of importance to understanding the influence of the pharmaceutical industry. The current study makes use of digital methods to estimate the number of MECCs, and to provide broad insights on this overlooked industry.

## MATERIALS AND METHODS

**Figure 1:**
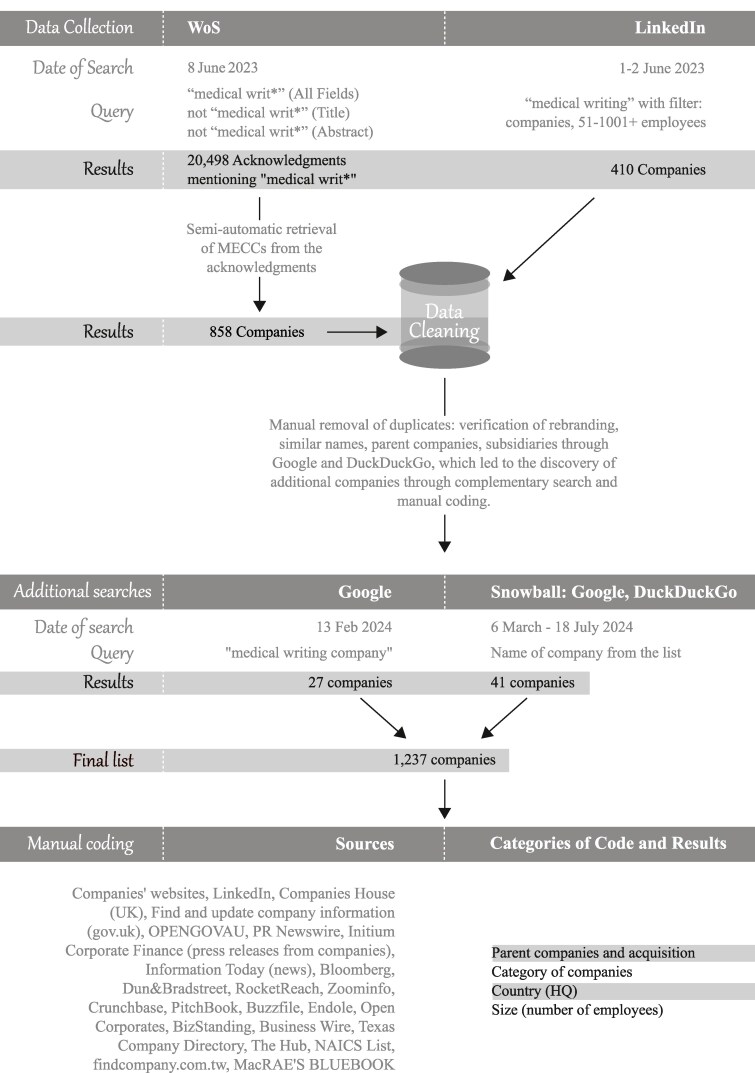
Procedures used to build and code the database.

The goal of this study is to estimate the number of MECCs and comparable entities contributing to the medical science literature in English. Because of the constant reshaping of this industry through rebranding, merging and dissolution, making an exhaustive list of MECCs and kindred companies is impossible. As Fig. 1 illustrates, our data set is a combination of data collected from Web of Science (WoS), LinkedIn, Google, and DuckDuckGo searches. We chose WoS because it yielded a much higher number of results than did PubMed, better fitting our goal of achieving as comprehensive coverage as possible. To extract companies mentioned in the paper acknowledgements, we used Excel functionality. We then supplemented that list of companies with lists collected from the more qualitative searches. Manually coding each MECC (e.g. the location of its headquarters, the number of employees, the core specialities of the company), meant following links that added data, leading to a snowball-style discovery of companies such as subsidiaries, parent companies, or companies that share similar names.

Because the search queries were in English, collected papers were published in English, and company names and information presented in English (LinkedIn, Google, and DuckDuckGo). Another limitation to the list of companies stems from the lack of transparency: For example, WoS only provides results for companies that are transparent about medical writing, which certainly misses some MECCs. The combination of different searches, plus collecting records of parent and subsidiary companies, helped to overcome this limitation. We also tried coding the data with the D&B Hoovers business database and analytical tool to gain insight on the companies we found, but it did not deliver results as current as our other methods.

## RESULTS

The WoS search produced 20 498 published papers mentioning medical writing in the acknowledgements (filtered in R with dplyr 1.1.4). Of these, 99.9% were published since 2008, a period during which the medical writing industry has been discussing concerns about transparency (Matheson 2019), and during which corporate integrity agreements governed some pharmaceutical companies’ publication planning (Rodino 2013); these contexts have increased the acknowledgement of medical writing, though likely not its incidence. We excluded 42 ‘false positives’ in which articles made such claims as ‘no medical writing assistance was used’, and an additional 211 papers that did not contain ‘medical writ*’ in their acknowledgements. From the database of papers, we identified 858 MECCs and other companies and institutions providing medical writing services.

The LinkedIn search produced 410 results, from which we identified 311 new companies. Our Google search identified another 27 companies, and the snowball search (searches on search engines of already found companies in WoS that led to the discovery of new companies) a further 41. Whereas the WoS search was not temporally bounded, the LinkedIn and Google searches were, necessarily, over small finite periods (see Fig. 1).

That these companies are engaged in publication planning was clear from their descriptions and listed specialities on LinkedIn and on their websites. The MECC with the largest contribution (975 publications) to the WoS results, inScience Communications (part of Springer Nature Group), was at the time of the search advertising for a position involving contributions to publication plans on LinkedIn. Websites of the MECCs identified publication planning as a core competence of 542 companies. The 89 LinkedIn job advertisements we analysed often described medical writer positions as involving liaison between internal and external stakeholders, or clients and KOLs, strongly suggesting that the work involved writing articles on behalf of sponsoring companies for authorship by individuals. For example, IQVIA required a senior scientific advisor to ‘[m]anage faculty recruitment, engagement, and relationships on behalf of clients’. We also note that 70% of entities in our database were obtained through direct acknowledgment of medical writing or medical writers within published articles. This was in the form of sentences very much like the one with which we began this paper, usually recognizing the writer or writers, the MECC involved, and the pharmaceutical company providing the funding.

Together, these searches produced 1237 entities of diverse kinds, sizes, locations, and offering different core services. Because of the dynamic nature of business, some have merged or dissolved, making their identification challenging.

**Figure A1.**
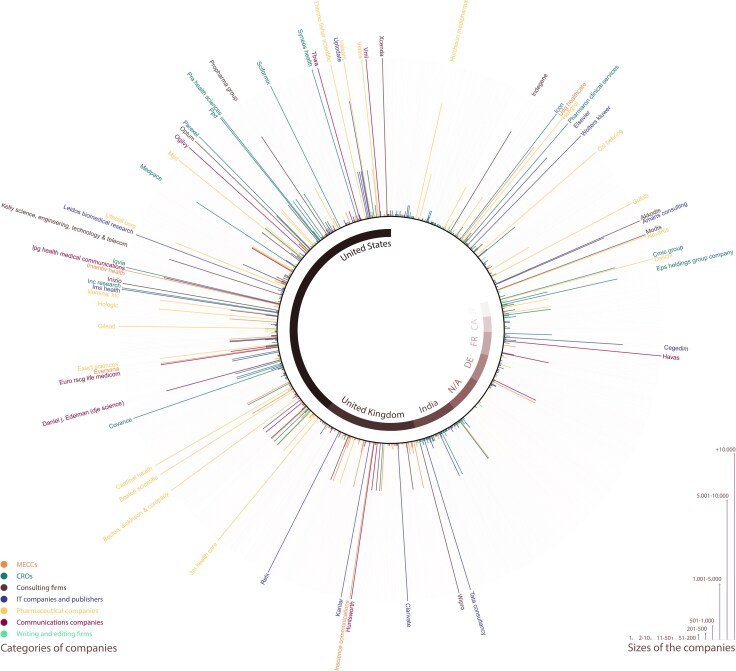
Visualization of the MECCs we found, by country, by type, and by size.

From our initial searches we removed a total of 89 hospitals and other healthcare institutions, universities, non-governmental organizations, professional associations, and miscellaneous other entities that were difficult to classify. We removed these either because they were not commercial or because they were different enough from MECCs and kindred entities that they stood a high chance of not being comparable to the rest of the list. As illustrated in Fig. A1, the result was 1148 MECCs or related companies offering medical writing services, with the following diverse core of identities:

**Table:** 

MECCs	542
Contract research organizations	243
Consulting firms	113
IT companies and publishers	82
Pharmaceutical companies	79
Communication companies	45
Writing and editing firms	44

Ten firms wrote 26% of the papers in our WoS papers database—omitting pharmaceutical companies (or their branches) engaged in medical writing, as we could not mechanically dissociate them from funders in the acknowledgements.

Table 1 is evidence that the bulk of medical writing services for papers are purchased by pharmaceutical and device companies. Strikingly, just ten pharmaceutical companies were reported as sponsors 12 512 times in WoS papers acknowledging medical writing support. Most of the remaining WoS papers in our database are funded by other pharmaceutical, device, and biotechnology companies.

**Table 1.** Top 10 MECCs and funders found in our database.

(a) Top 10 MECCs or equivalents acknowledged as providing medical writing services in our database, and citations of their papers (data extracted from WoS). Not included are pharmaceutical companies providing medical writing services.			
Companies	Papers	Times cited (WoS)	Times cited per paper
Springer Healthcare/inScience Communications	**975**	21 997	**22.56**
Ashfield	**887**	26 074	**29.40**
Complete Medical Communications/CMC Affinity/ CMC Connect	**623**	18 285	**29.35**
Peloton Advantage	**491**	10 170	**20.71**
Engage Scientific	**454**	10 438	**22.99**
Envision Scientific Solutions/Envision Pharma Group	**413**	9257	**22.41**
UDG Healthcare	**407**	13 259	**32.58**
Oxford PharmaGenesis	**384**	9800	**25.52**
Complete Healthcare or CHC Group	**322**	12 477	**38.75**
Parexel	**295**	10 451	**35.43**

(b) Top 10 companies acknowledged as funders of the papers in our database (data extracted from WoS). Not included in these numbers are papers by acquired companies with different names. Some papers are funded by more than one company.			
Pfizer	**2412**	71 619	**29,69**
Novartis	**1661**	61 269	**36,89**
AstraZeneca	**1577**	59 265	**37,58**
Merck	**1410**	57 508	**40,79**
Roche	**1063**	49 738	**46,79**
Sanofi	**1043**	34 540	**33,12**
AbbVie	**864**	18 818	**21,78**
Boehringer Ingelheim	**862**	41 398	**48,03**
Eli Lilly	**817**	28 691	**35,12**
Bristol Myers Squibb	**803**	48 858	**60,84**

In Table 1 we included the numbers of citations to the papers captured. These numbers are well above average, suggesting that this funded literature has a substantial impact. Potential explanations include the involvement as authors of KOLs who are prominent scientists in their fields, and/or self-citations and cross-citations among papers produced for a pharmaceutical company, and/or circulation of papers by companies and their agents. However, further investigations would be necessary to explain high citation rates.

## DISCUSSION

There is a sizeable medical writing industry that has attracted very little attention in the scientific literature. Working from more than 20 000 articles in the WoS database acknowledging medical writing, from more than 400 company profiles on LinkedIn mentioning medical writing, and filling in gaps through other searches, we found 1148 companies offering medical writing services. Medical writing for the pharmaceutical industry has become normalized.

There are reasons why authors might employ medical writers to help with scientific papers, including the challenges of coordinating multiple people, of writing in English, and of addressing the requirements of journals (e.g. Langdon-Neuner 2008). However, from the WoS data we see that most MECCs and other firms we catalogued earn their medical writing revenue not by working for individual authors but by working for industry, which creates the financial potential of this market.

Our results also show funders of the medical writing literature are prominently pharmaceutical companies. In multiple studies, pharmaceutical industry funding has been shown to influence published results and conclusions (see Lundh *et al*. 2017). Pharmaceutical companies and their service providers can deliver, and can thus control and influence, every step of the research process, and the analysis, writing, and publication of papers, in ways that are opaque to readers and to some involved in the process as well. These companies not only fund research, but also routinely design and shape it. They hire contract research organizations to conduct the bulk of their funded research, especially clinical trials (Mirowski and Van Horn 2005); sometimes those contract research organizations also offer medical writing and similar services, integrating many steps in the production of medical information. MECCs hire medical writers to produce drafts and revisions of papers, and those companies shepherd manuscripts through the writing and publication process (Sismondo 2018). MECCs can also be specialized in marketing and sometimes legal and public relations, and those services can be interwoven with medical writing. Vertical integration, whether in companies primarily seen as contract research organizations or as MECCs or as pharmaceutical companies, allows information to be neatly shaped and packaged for particular audiences—from researchers to patients—and purposes.

Through these actions, pharmaceutical companies contribute to the relevant medical science literature, perhaps to affect the general knowledge base and the landscape on which prescribers make decisions, and perhaps to be able to draw on specific claims in targeted sales efforts: using ghosted papers, pharmaceutical companies can steer scientific arguments in favour of their new products, recruiting KOLs to serve as their mouthpieces; they can direct attention to health concerns that are profitable to them, but not necessarily of intrinsic importance or of medical value to the public; companies can hype their products at the expense of older, less expensive products; they can shape perceptions of risks and benefits, with subtle and not-so-subtle rhetorical and statistical spin; they can produce evidence that feeds into evidence-based medicine; and companies can protect their investments when prescribers and publics question the efficacy and safety of specific products (e.g. Michaels 2020, Bernisson and Sismondo 2024). Given this infrastructure of influence, medical writing firms are neither independent researchers nor peripheral actors, but form a core part of how sponsored knowledge is constructed and disseminated. Thus, we should care about the size of the medical writing industry.

## Data Availability

The data that support the findings of this study are available on request from the corresponding author, M.B.
